# Formation of new lymphatic vessels in glioma: An immunohistochemical analysis

**DOI:** 10.1111/neup.12625

**Published:** 2020-01-20

**Authors:** Fan‐Wei Meng, Fu‐Sheng Liu, Wen‐Hui Liu, Li Li, Lin‐Lin Jie

**Affiliations:** ^1^ Department of Anatomy and Physiology Shandong College of Traditional Chinese Medicine Yantai China; ^2^ Department of Neurosurgery Beijing Tiantan Hospital Affiliated to Capital Medical University, Beijing Neurosurgical Institute Beijing China

**Keywords:** glioma, hypoxia‐inducible factor 1α, lymphatic endothelial hyaluronic acid receptor 1, lymphatic vessels, Prospero‐Related Homeobox 1

## Abstract

We investigated the distribution and formation of new lymphatic vessels in gliomas. Specimens from seven glioma cases were analyzed by immunohistochemical staining for CD34, lymphatic endothelial hyaluronic acid receptor 1 (LYVE‐1), prospero‐related homeobox 1 (Prox1), nestin, and hypoxia‐inducible factor 1α (HIF‐1α). Three types of vessels were observed in glioma specimens: LYVE‐1^+^ lymphatic vessels, CD34^+^ blood vessels, and LYVE‐1^+^/CD34^+^ blood vessels. Prox1^+^/LYVE‐1^+^ cells were distributed in some lymphatic vessels as well as among vascular endothelial cells and glioma cells. Nestin^+^ cells were scattered throughout the gliomas, and some lymphatic cells also expressed nestin. HIF‐1α^+^ Prox1^+^ cells were widely distributed within the glioma specimens. The present immunohistochemical analysis revealed upregulation of Prox1 and HIF‐1α in some glioma tissues as well as the differentiation of nestin^+^ tumor stem cells into LYVE‐1^+^ lymphatic vessels.

## INTRODUCTION

Glioma is the most common primary central nervous system (CNS) tumor, accounting for approximately 31% of all CNS tumors in the USA and 81% of all malignant CNS tumors.^1^, ^2^ There is considerable heterogeneity among the different types of glioma, and glioblastoma (GBM) has the highest incidence rate.^3^ Survival times vary among glioma patients according to the different grades of glioma, and the prognosis is worst for patients with GBM, which is associated with a 5‐year survival rate of less than 5%.^2^ GBM also has a high probability of recurrence, and even with successful initial surgical resection, almost all GBM patients experience tumor recurrence.^4^, ^5^, ^6^

Nedergaard et al. first discovered how cerebrospinal fluid and brain tissue fluids are exchanged and named the lymphatic pathway in 2012.^7^ Kipnis et al. identified the meningeal lymphatic vessels via staining of the whole meninges and revealed the presence of lymphatic endothelial cells, confirming the existence of lymphatic vessels in the brain.^8^ However, whether glioma tissues contain lymphatic vessels remains unknown.

In the present study, we examined the presence and distribution of new lymphatic vessels in glioma samples from seven patients via immunohistochemical staining for the neovascular endothelial cell marker molecule CD34, lymphatic endothelial hyaluronic acid receptor 1 (LYVE‐1), prospero‐related homeobox 1 (Prox1), the neuroepithelial stem cell protein nestin, and hypoxia‐inducible factor 1α (HIF‐1α). The presence of lymphatic vessels within glioma tissues may offer a new strategy for the treatment of glioma.

## MATERIALS AND METHODS

### Materials

Goat anti‐human LYVE‐1 antibody (R&D Systems, Minneapolis, MN, USA), fluorescence‐labeled rabbit anti‐goat antibody (Antigen, Wuhan, China), rabbit anti‐human Prox1 antibody (Boster Biological Technology, Wuhan, China), rabbit anti‐human nestin antibody (Boster), and rabbit anti‐human HIF‐1α (Boster Biological Technology Co. Wuhan, China) were used as the primary antibodies for immunohistochemistry. Immunoreaction product deposits were visualized with a 3,3′‐diaminobenzidine (DAB) immunostaining kit (Boster Biological Technology) along with 10 mM phosphate‐buffered saline (PBS), pH 6.0.

### Specimen preparation

Glioma specimens were obtained from the Department of Neurosurgery of Beijing Tiantan Hospital (five cases) and the Department of Neurosurgery of Binzhou Medical College (two cases) (Table 1). The use of these specimens was approved by the ethics committee of Shandong College of Traditional Chinese Medicine. The excised tumor tissues were fixed in 40 g/L formaldehyde and embedded in paraffin for sectioning at a thickness of 4 μm. Hematoxylin and eosin (HE) staining was applied to observe the histomorphology of the tumor tissues. Tissues adjacent to the glioma, which were pathologically confirmed to be devoid of tumor cells, were used as normal control specimens.

**Table 1:** Clinical characteristics of seven patients with glioma and one control

Patient no.	Sex	Age (years)	Histological diagnosis	Pathological WHO grade	Treated with bevacizumab
1	Female	62	Glioblastoma	IV	No
2	Female	44	Astrocytoma	II	No
3	Male	19	Astrocytoma	II	No
4	Female	45	Anaplastic astrocytoma	III	No
5	Female	40	Anaplastic astrocytoma	III	No
6	Male	54	Glioblastoma	IV	No
7	Male	63	Glioblastoma	IV	No
8	Male	18	Normal brain tissue	Control (surgically treated for injuries from traffic accident)	No

### Immunohistochemical analysis

After the tissue sections were deparaffinized, antigen retrieval was achieved by immersion in 0.01 mol/L citrate buffer at 121°C for 15 min, and endogenous peroxidase activity was blocked with 0.3% hydrogen peroxide. For immunohistochemistry, the sections were incubated for 24 h at at 4C in solutions of rabbit anti‐human CD34 antibody (dilution 1:200), rabbit anti‐human Prox1 antibody (1:100), rabbit anti‐human HIF‐1α antibody 1:100), and goat anti‐human LYVE‐1 antibody (1:100). Next the sections were incubated with horseradish peroxidase (HRP)‐labeled goat anti‐rabbit immunoglobulin G (IgG) or rabbit anti‐goat IgG, and finally, DAB staining was performed. All stained sections were observed and photographed at a magnification of 40× under a fluorescence microscope (DP‐72, Olympus). Semi‐quantitative analysis was performed using Image‐Pro Plus 6.0, and the average optical density values were compared using *t*‐test with SPSS statistical software (SPSS Inc., Chicago, IL, USA). Values of *P* < 0.05 were considered statistically significant.

After reactivation of antigen within the paraffin‐embedded sections, the sections were incubated with the primary antibodies (1:100) at 4°C for 24 h. Then the sections were incubated with Cy3‐labeled donkey anti‐goat IgG or fluorescein isothiocyanate (FITC)‐labeled donkey anti‐rabbit IgG, rabbit anti‐mouse nestin (1:100), goat anti‐mouse LYVE‐1 (1:100), and FITC‐labeled donkey anti‐goat IgG or Cy3‐labeled donkey anti‐rabbit IgG (1:100). Finally, the sections were observed under a DP‐72 fluorescence microscope.

## RESULTS

### Histomorphology of glioma tissues

**Figure 1:**
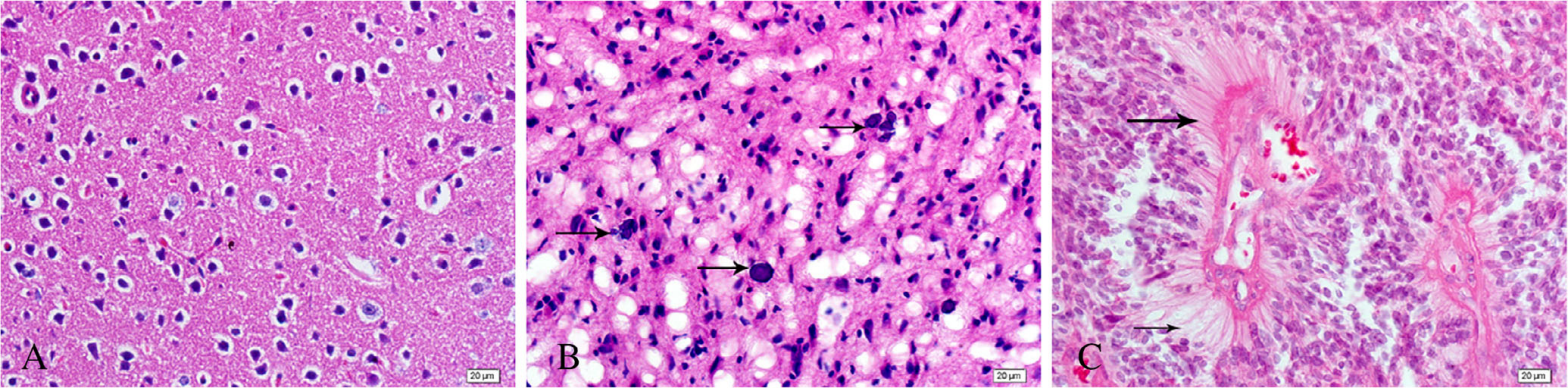
Representative images of HE staining of a normal brain tissue (A) and glioma tissues (B and C). The arrows indicate pleomorphism of the cell nuclei (B) and neovascularization and necrosis in the tumors (C). In the normal brain tissue, cells are arranged regularly, and the blood vessels are clear. The nuclei of the glioma cells are irregular in a vigorously dividing state, and necrosis is observed in some areas. Scale bars: 20 μm (A‐C).

Compared with normal brain tissue (Fig. 1A), glioma tissues exhibited more nuclear pleomorphism, and divided nuclei were observed in glioma cells (Fig. 1B). HE staining also revealed abundant neovascularization within the tumor tissue, and necrosis was obvious (Fig. 1C).

### Blood and lymphatic vessels within glioma tissues

**Figure 2:**
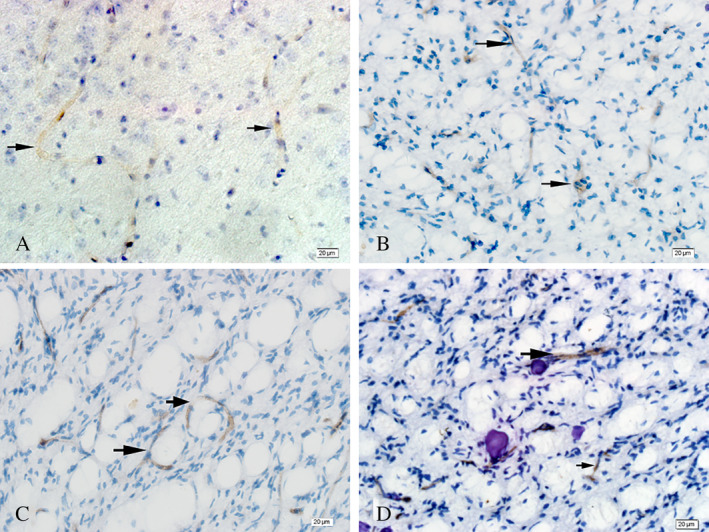
CD34 immunohistochemistry in a normal brain tissue (A) and glioma tissues (B‐D). The endothelial cells and walls of blood vessels in the normal brain tissue are clear and intact. The vascular walls in the glioma samples are rough with an unclear boundary between the vessels and surrounding tissue. Scale bars: 20 μm (A‐D).

**Figure 3:**
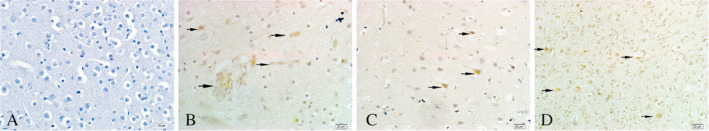
LYVE‐1 immunohistochmistry in a normal brain tissue (A) and glioma tissues (B, C). The arrows indicate LYVE‐1^+^ cells. No LYVE‐1^+^ cells are observed in normal brain tissue, unlike in gliomas where many LYVE‐1^+^ ‐positive cells are observed, with some forming tubular structures. Scale bars: 20 μm (A‐D).

**Figure 4:**
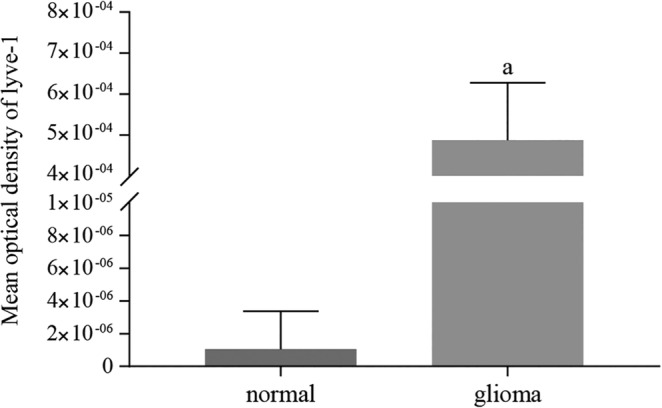
Mean optical density of LYVE‐1 immunohistochemistry in normal brain and glioma tissues. LYVE‐1 staining is significantly greater in the glioma tissue compared with the normal brain tissue.

**Figure 5:**
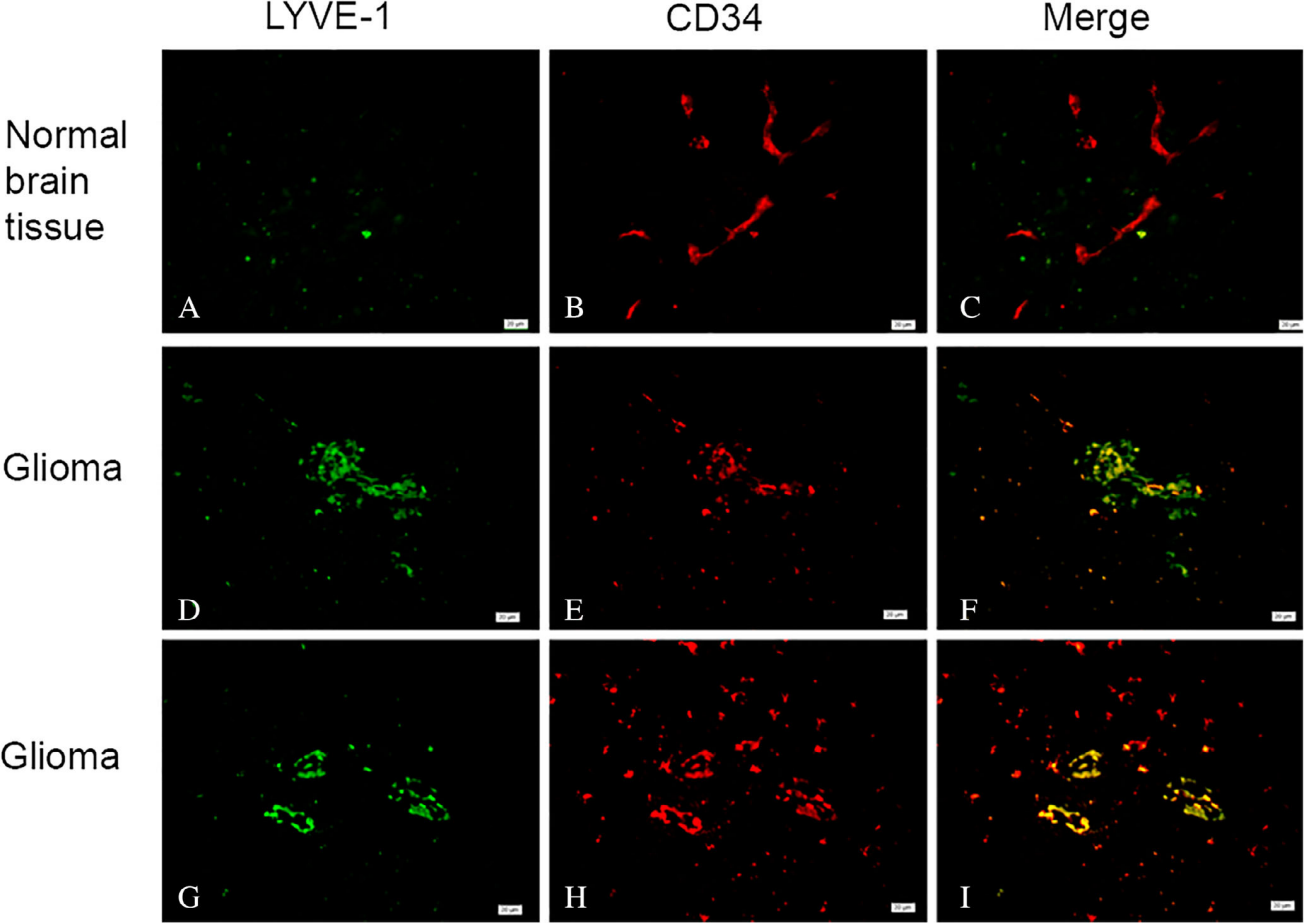
Double‐labeling immunofluorescence staining for LYVE‐1 (green) and CD34 (red) in a normal brain tisue (A‐C) and glioma tissues (B‐G). Many LYVE‐1^+^ / CD34^+^ cells and tubular structures are observed in the glioma tissues. Scale bars: 20 μm (A‐I).

As shown in Figure 2A, capillary walls consisting of CD34^+^ cells were completely continuous, and the corresponding lumen was dilated in normal brain tissue. Although linear capillaries (Fig. 2A, B, D) and circular tubular structures (Fig. 2C) formed by CD34^+^ cells were observed in the glioma samples, the associated lumen was thin and the tube wall was rough. No LYVE‐1 staining was observed in normal brain tissue (Fig. 3A), but LYVE‐1^+^ cells were found in glioma samples where they were mainly distributed among small blood vessels and capillary endothelial cells (Fig. 3B). Moreover, strong positive staining for LYVE‐1 was observed in some tumor cells (Fig. 3C). The average optical density value for LYVE‐1^+^ staining was significantly greater in glioma tissue than in normal tissue (Fig. 4). Additionally, many vessels formed by LYVE‐1^+^/CD34^+^ cells were seen in the gliomas (Fig. 5F, I), along with many red blood cells (Fig. 5F, I).

### Lymphatic endothelial cells in glioma tissues

**Figure 6:**
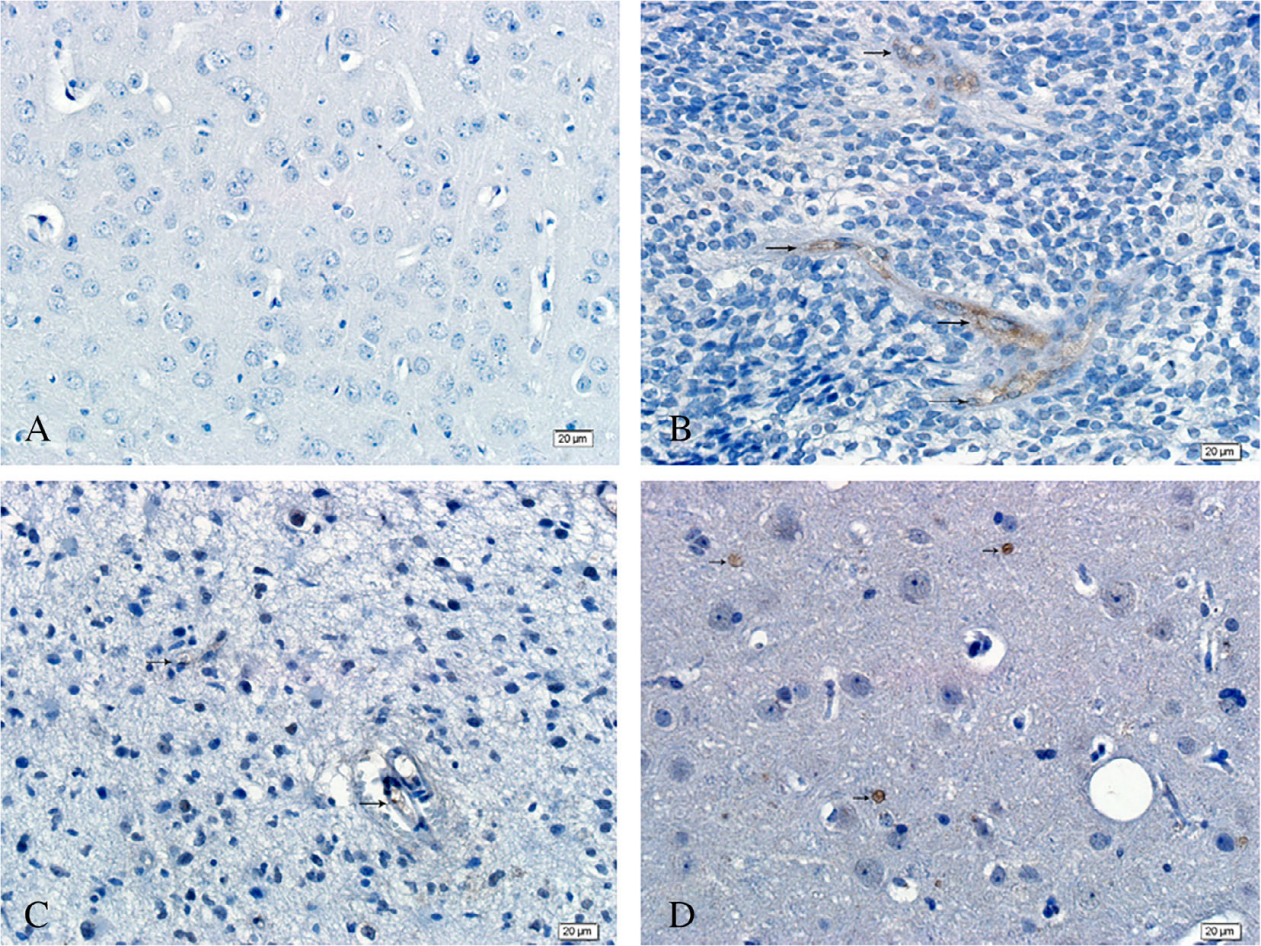
Prox1 immunohistochemistry in a normal brain tissue (A) and glioma tissues (B‐D). The brown color indicates staining for Prox1. No Prox1 immunoreactivity is observed in the normal brain tissue, but the immunoreactivity is observed in some vascular endothelial cells in the glioma tissues. Scale bars: 20 μm (A‐D).

**Figure 7:**
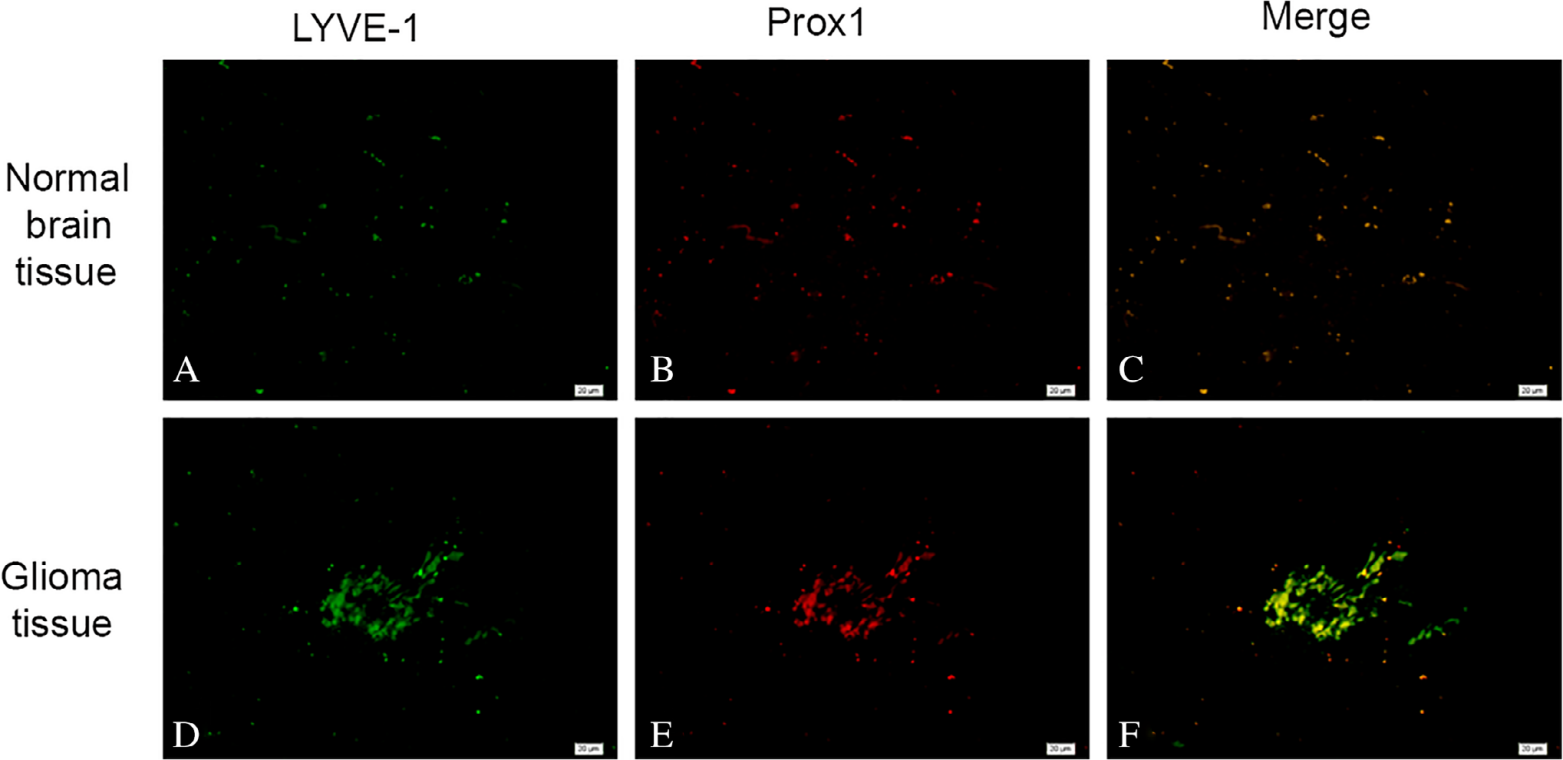
Double‐labeling immunofluorescence staining for LYVE‐1 (green) and Prox1 (red) in normal brain tissues (A‐C) and glioma tissues (D‐F). In the glioma tissues, some cells within tubular structures express both Prox1‐ and LYVE‐1‐specific markers of lymphatic endothelial cells. Scale bars: 20 μm (A‐F).

No Prox1^+^ cells were observed in the normal tissues (Fig. 6A), whereas Prox1^+^ cells were distributed in large and small blood vessels and among capillary endothelial cells in gliomas (Fig. 6B–D). Staining for Prox1 revealed abundant LYVE1^+^/Prox1^+^ tumor cells (Fig. 7C) and lymphatic endothelial cells (Fig. 7F) in gliomas.

### Neuroepithelial stem cells in glioma tissues

**Figure 8:**
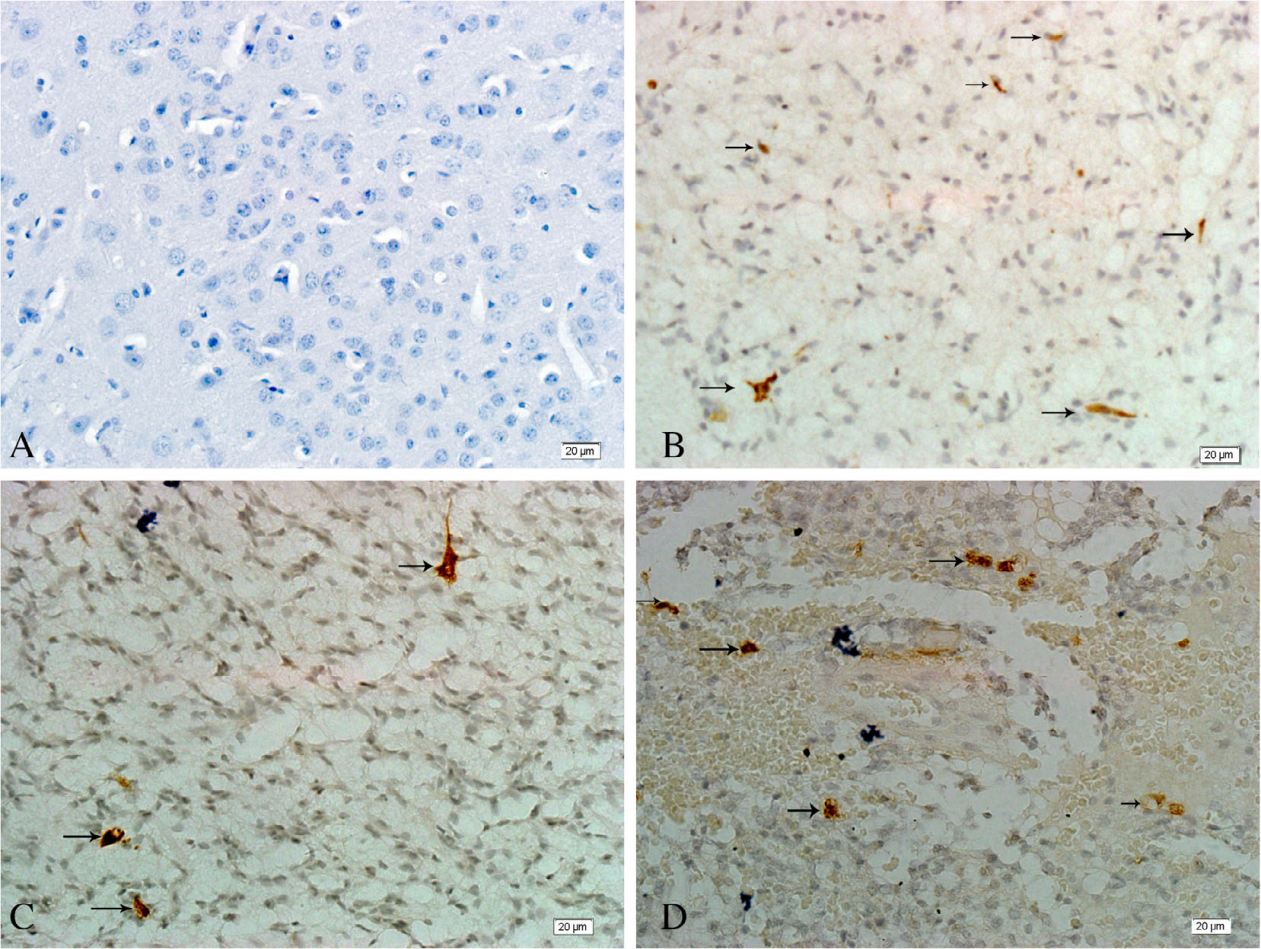
Nstin immunohistochemistry in a normal brain tissue (A) and glioma tissues (B‐D). The arrows indicate nestin^+^ cells. No nestin^+^ cells are observed in the normal brain tissues, whereas nestin^+^ cells are seen in most of the glioma tissues. Scale bars: 20 μm (A‐D).

**Figure 9:**
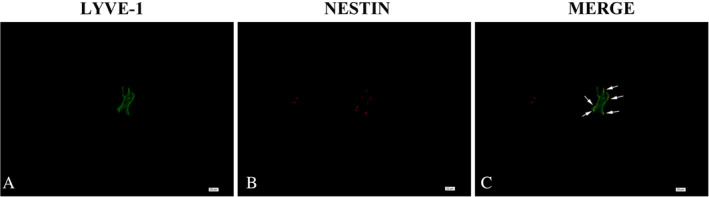
Double‐labeling immunofluorescence staining for LYVE‐1 (green) and nestin (red) in glioma tissues. These images are from a tumor of pathological grade 2. Complete lymphatic vessels with LYVE‐1^+^ cells are observed in the glioma tissues, and some endothelial cells express nestin. Scale bars: 20 μm (A‐C).

No nestin^+^ cells were observed in normal brain tissue (Fig. 8A), whereas nestin^+^ cells were scattered within the glioma tissues (Fig. 8B), with some forming a capillary‐like vessel structure (Fig. 8C, D). The nestin^+^ cells were distributed among LYVE‐1^+^ lymphatic endothelial cells (Fig. 9D), and many LYVE‐1^+^ nestin^+^ tumor cells were identified.

### Hypoxia among glioma cells

**Figure 10:**
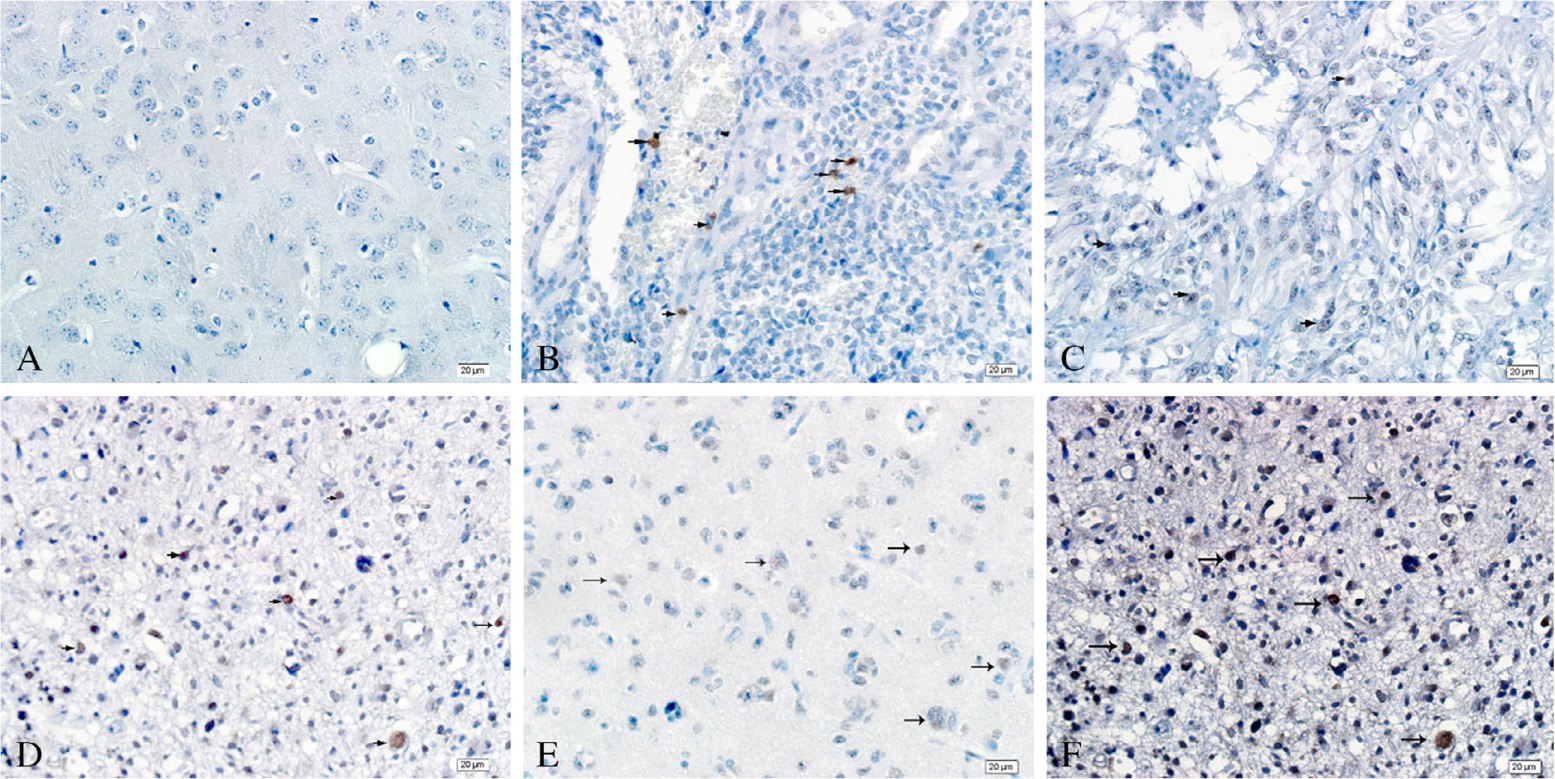
HIF‐1α immunohistochemistry in a normal brain tissue (A) and glioma tissues (B‐F). The brown color and arrows indicate HIF‐1α^+^ staining. No HIF‐1α expression is observed in the normal brain tissue, whereas HIF‐1α^+^ cells are distributed in most of the glioma tissues. Scale bar: 20 μm (A‐F).

**Figure 11:**
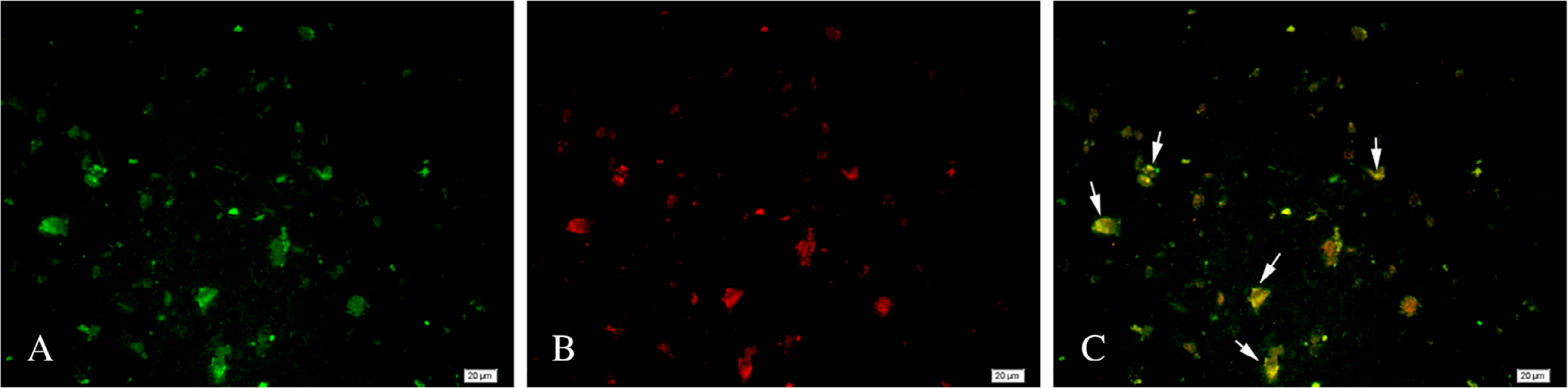
Double‐labeled immunofluorescence staining for Prox1 (green) and HIF‐1α (red) in glioma tissues. Many Prox1^+^ / HIF‐1α^+^ cells are observed in the glioma tissues. Scale bars: 20 μm (A‐C).

No HIF‐1α^+^ cells were observed in normal brain tissue (Fig. 10A). In contrast, HIF‐1α^+^ cells were distributed in glioma samples, but their number and staining intensity varied among the samples (Fig. 10B–F). Notably, many Prox1^+^ HIF‐1α^+^ cells were distributed in gliomas but not seen in normal brain tissue (Fig. 11).

## DISCUSSION

The present immunohistochemical analysis of glioma tissues from seven patients revealed the presence of new lymphatic vessels in gliomas. Tumor lymphangiogenesis is induced by HIF‐1α expression and mediated by interactions between vascular endothelial growth factor (VEGF) in tumor cells^9^, ^10^ and macrophages^11^, ^12^ and VEGF receptors (VEGFRs) on existing vascular endothelial cells,^13^ lymphatic endothelial cells,^14^ and macrophages in the tumor microenvironment.^15^ LYVE‐1 is an endothelial cell receptor that plays a critical role in dendritic cell entry into lymphatic vessels, and deletion of LYVE‐1 reduces CD8^+^ T‐cell responses in skin‐draining lymph nodes.^16^ In the present study, we found that LYVE‐1 was highly expressed in some glioma cells, which is consistent with a previous report that *LYVE‐1* is a hypoxia‐regulated gene and its expression is significantly negatively associated with glioma patient survival.^17^

Prox1 expression has been found in many tumor types, including glioma.^18^, ^19^ Here, we found that Prox1 was highly distributed in endothelial cells of blood vessels and capillaries within glioma tissues. Previous studies demonstrated that the percentage of Prox1^+^ cells increases with tumor grade in astrocytic brain tumors and that Prox1 can serve as a prognostic indicator in high‐grade gliomas.^20^, ^21^ Nestin is also a marker of glioma and may play an important role in the prediction of the clinicopathology and prognosis of glioma patients.^22^, ^23^ Consistently, we found scattered nestin^+^ cells in the glioma samples.

Growing gliomas have a hypoxic microenvironment, and HIF‐1α expression has been found to be increased in *isocitric dehydrogenase (IDH)* mutant gliomas.^24^ However, other studies have indicated that HIF‐1α expression is either not affected by an *IDH* mutation or even down‐regulated.^25^, ^26^ Research has also shown that higher HIF‐1α expression is associated with a higher degree of tumor malignancy.^27^ In the present study, we detected HIF‐1α expression at variable levels in different glioma specimens. However, HIF‐1α expression was significantly increased in glioma tissues compared with normal brain tissues.

Finally, we examined the association between HIF‐1α and the key factor in lymphangiogenesis, Prox1, and showed that HIF‐1α^+^ tumor cells simultaneously expressed Prox1. Therefore, it can be concluded that rapid proliferation of glioma cells triggers hypoxia within the tumor cells, activating downstream genes including *HIF‐1α* and *Prox1* and leading to tumor cell differentiation and expression of LYVE‐1. Such differentiated LYVE‐1^+^ cells then associate to form lymphatic vessels.

Previous reports have found that proteins that are typically involved in lymphangiogenesis are expressed in glioma.^28^, ^29^ Grau et al. described a lymphatic phenotype of tumor vessels in malignant gliomas and glioblastomas as well as endothelial expression of VEGFR3 in the entire tumor vasculature with a high expression of podoplanin in cell scaffolding vessel structures.^30^ Jiang et al. detected the expression of lymphangiogenesis markers in primary and recurrent glioma tumors.^31^ At present, the source of new lymphatic vessels in tumors remains controversial. Some researchers believe that lymphatic vessels are formed within tumors by regeneration of original lymphatic vessels and then induce metastasis.^32^ Others believe that lymphatic vessels can form independently in tumors and around tumors, leading to metastasis.^33^ Previous findings have confirmed the generation of lymphatic vessels in breast cancer.^34^, ^35^ Together these results suggest that lymphatic vessels formed by invasive cancer cells can provide access to the lymphatic system to accelerate lymphatic metastasis.

In summary, we observed up‐regulated co‐expression of Prox1 and HIF‐1α in gliomas as well as the differentiation of nestin^+^ tumor stem cells into LYVE‐1^+^ lymphatic endothelial cells that formed lymphatic vessels. Our study provides the first demonstration of lymphatic vessels in gliomas.

## AUTHOR CONTRIBUTIONS

Conceptualization, FWM; methodology, FWM; software, FWM; validation, FSL; formal analysis, FWM; investigation, FWM; resources, WHL; data curation, FWM; writing – original draft preparation, FWM; writing – review and editing, FSL; project administration, LL; funding acquisition, LLJ.

## DISCLOSURE

The authors declare they have no conflict of interest.
